# Changes in Stress Urinary Incontinence Symptoms after Pelvic Organ Prolapse Surgery: a Nationwide Cohort Study (FINPOP)

**DOI:** 10.1007/s00192-024-05760-0

**Published:** 2024-03-28

**Authors:** Päivi K. Karjalainen, Anna-Maija Tolppanen, Olga Wihersaari, Kari Nieminen, Nina K. Mattsson, Jyrki T. Jalkanen

**Affiliations:** 1 Department of Obstetrics and Gynecology, Wellbeing Services County of Central Finland/Hospital Nova of Central Finland, Hoitajantie 3, 40620 Jyväskylä, Finland; 2 Institute of Clinical Medicine, University of Eastern Finland, Kuopio, Finland; 3 School of Pharmacy, University of Eastern Finland, Kuopio, Finland; 4 Faculty of Medicine and Health Technology, Tampere University, Tampere, Finland; 5 Department of Obstetrics and Gynecology, Tampere University Hospital, Tampere, Finland; 6 Aava Medical Centre, Helsinki, Finland; 7 Wellbeing Services County of Central Finland, Jyväskylä, Finland

**Keywords:** De novo stress urinary incontinence, Pelvic organ prolapse, Stress urinary incontinence, Prognostic factor, Pelvic organ prolapse surgery

## Abstract

**Introduction and hypothesis:**

Various strategies are employed to manage stress urinary incontinence (SUI) during pelvic organ prolapse (POP) surgery. This study was aimed at facilitating shared decision-making by evaluating SUI symptom changes, staged SUI procedures, and their prognostic factors following POP surgery without concomitant SUI intervention.

**Methods:**

We analyzed 2,677 POP surgeries from a population-based observational cohort, excluding patients with prior SUI surgery. The outcome measures were subjective SUI utilizing the Pelvic Floor Distress Inventory-20 questionnaire and number of subsequent SUI procedures. Multivariable linear models were applied to identify predictors of persistent SUI, procedures for persistent SUI, and de novo SUI. The primary assessment occurred at the 2-year follow-up.

**Results:**

At baseline, 50% (1,329 out of 2,677) experienced SUI; 35% (354 out of 1,005) resolved, an additional 14% (140 out 1,005) improved, and 5.1% (67 out of 1,308) underwent a procedure for persistent SUI. De novo SUI symptoms developed in 20% (218 out of 1,087), with 3.2% (35 out of 1,087) reporting bothersome symptoms; 0.8% (11 out of 1,347) underwent a procedure for de novo SUI. High baseline symptom severity increased the risk of persistent SUI (adjusted odds ratio [aOR] 2.04, 95% confidence interval [CI] 1.65–2.53), whereas advanced preoperative apical prolapse decreased the risk (aOR 0.89, 95% CI 0.85–0.93). De novo SUI was more common with advancing age (aOR 1.03, 95% CI 1.01–1.05), baseline urgency urinary incontinence (aOR 1.21, 95% CI 1.06–1.38), and after transvaginal mesh surgery (aOR 1.93, 95% CI 1.24–3.00). It was not dependent on the compartment or preoperative degree of prolapse.

**Conclusions:**

In a pragmatic setting, POP surgery results in a low rate of subsequent SUI procedures.

## Introduction

Half of women undergoing pelvic organ prolapse (POP) surgery report pre-existing stress urinary incontinence (SUI), which may persist or resolve after POP surgery [1]. Moreover, up to half of continent women develop new-onset SUI postoperatively [1–5]. Randomized controlled trials (RCTs) indicate that combining prolapse surgery with a continence procedure can reduce postoperative SUI risk but at the expense of increased adverse events [6].

Global POP surgery practices vary, from routinely combining SUI procedures with POP surgery, irrespective of continence status, to avoiding concomitant SUI procedures, even in women with SUI. Treating all women with a combination of POP and SUI surgery might prevent subsequent SUI procedures but could also entail risks of overtreatment and unnecessary morbidity. A selective strategy targeting concomitant SUI procedures for women at risk of postoperative SUI faces challenges due to clinicians’ limited ability to accurately predict who benefits. In a staged strategy, continence surgery is performed secondarily after prolapse surgery only when necessary, avoiding unnecessary procedures but exposing some women to two operations.

Women need valid information about the benefits and harms of each option to make informed decisions regarding SUI strategy during their POP surgery. Although RCTs offer relative risks between combination surgery and the staged strategy, their absolute risk estimates may not apply to everyday practice as their protocols differ from typical clinical scenarios.

The primary objective of this study was to describe changes in SUI symptoms after POP surgery in a pragmatic setting, providing realistic expectations for surgeries without concomitant SUI procedures. The secondary objective was to identify prognostic factors for persistent and de novo SUI symptoms, exploring ways of selecting patients for concomitant SUI surgery.

## Materials and Methods

### Setting and Participants

We utilized data from the Finnish Pelvic Organ Prolapse Surgery Survey (FINPOP), a nationwide prospective observational cohort study covering 83% (*n* = 3,535) of POP surgeries conducted in Finland between 1 January and 31 December 2015 (*n* = 4,240). Among the 45 Finnish hospitals performing POP surgery, 41 recruited all women scheduled for POP surgery during the timeline, excluding those unable to communicate in Finnish or Swedish. Surgical methods and decisions regarding continence procedures were determined by individual surgeons’ discretion [7]. The study was approved by the Research Ethics Committee of the Northern Savo Hospital District (reference number 5//2014) and adhered to the ethical principles of the Declaration of Helsinki. Each participant provided written informed consent.

For the present study, we included women who had completed the baseline symptom questionnaire, including the question about SUI symptoms. Women with a history of previous continence procedures were excluded. Women with concomitant continence procedures were part of the baseline assessment but were excluded from the follow-up data analysis.

### Data Collection

The surgeons completed standardized physician questionnaires at baseline, which involved recording the participants’ surgical history, operation details, and the degree of POP using the simplified Pelvic Organ Prolapse Quantification (POP-Q) [8]. The participants completed self-administered, standardized questionnaires at baseline, and at 6 and 24 months after surgery. These questionnaires included information on medical, surgical, and obstetric history, and the Pelvic Floor Distress Inventory−20 (PFDI-20) to assess pelvic floor symptoms. The PFDI-20 has been validated in Finnish and Swedish [9, 10].

Information on subsequent SUI procedures (surgery or periurethral bulking) during the follow-up period was obtained from the Care Register for Health Care, a national database that collects details about inpatient care, day surgeries, and specialized outpatient care. Both public and private hospitals are obliged to report information to this registry, which has a coverage rate of over 95%. The quality of this register in terms of completeness and accuracy is considered good [11].

### Outcome Measures, Data Handling, and Statistical Analyses

The primary outcome was subjective SUI measured using item 17 in the PFDI-20. The secondary outcome was number of subsequent SUI procedures after POP surgery. We considered the 24-month follow-up as our primary timepoint.

The scale for item 17 in the PFDI-20, “Do you usually experience urine leakage related to coughing, sneezing, or laughing?”, is as follows: 0—symptom not present; 1—symptom present but not at all bothersome; 2—symptom somewhat bothersome; 3—symptom moderately bothersome; 4—symptom quite a bit bothersome. We used the following definitions: baseline SUI was defined as a baseline score > 0; responses 3 and 4 were defined as bothersome; among the women with baseline SUI, SUI was defined as resolved if the follow-up score was 0, improved when the bother score decreased, persistent if the follow-up score was > 0, and worsened when the bother score increased; among the women without baseline SUI, follow-up scores 1–4 indicated de novo SUI of any degree and scores 3–4 indicated bothersome de novo SUI.

We describe changes in SUI symptoms between baseline and 6 and 24 months after surgery and report the number of subsequent SUI procedures during the 24-month follow-up period. Analyses related to symptom changes were conducted for cases with available follow-up symptom questionnaires, whereas analyses concerning subsequent SUI procedures were performed for the entire study population. For women who underwent a subsequent surgical procedure for SUI during the follow-up period, a bother score of 4 (indicating quite a bit bothersome) was assigned after the date of the SUI procedure. This approach was chosen, as our objective was to investigate whether women experienced SUI after POP surgery (i.e., not to assess the effects of the SUI procedure).

**Fig. 1:**
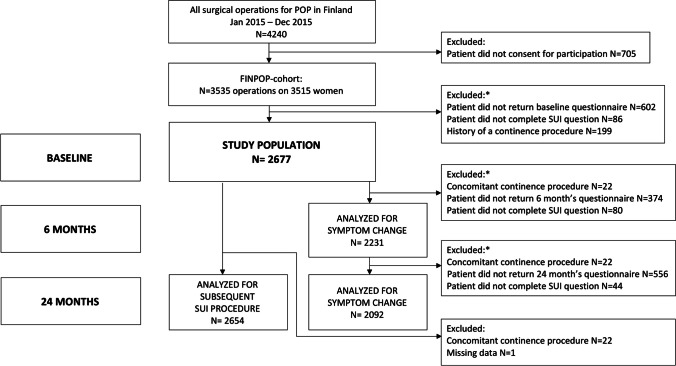
Flowchart showing the selection of the study population and the number of patients analyzed at different time points

To assess improvement in symptoms over time, we used the Wilcoxon signed rank test to compare baseline and 24-month bother scores among women with pre-existing SUI. To identify prognostic factors for baseline and postoperative SUI, we employed a generalized linear model with the ordinal bother score as the dependent variable. We used 24-month data for persistent SUI and 6- and 24-month data for de novo SUI. To examine prognostic factors for a procedure for persistent SUI during the 24-month follow-up period, we used a binary logistic regression model.

**Table 1:** Characteristics of the study population (*N* = 2,677)

Variable	Value	Data missing, *n* (%)
Age (years), mean ± SD	63.7 ± 10.5	2 (0.1)
BMI (kg/m^2^), mean ± SD	26.8 ± 4.1	86 (3.2)
POP-Q point Ba ≥ 0, *n* (%)	1,706 (66)	86 (3.2)
POP-Q point C ≥ 0, *n* (%)	1,057 (41)	121 (4.5)
POP-Q point Bp ≥ 0, *n* (%)	1,143 (44)	96 (3.6)
Vaginal parity, median (IQR)	2 (1)	59 (2.2)
Current smoker, *n* (%)	236 (8.9)	11 (0.4)
Diabetes, *n* (%)	249 (9.3)	0
Prior POP surgery, *n* (%)	651 (24)	0
Prior hysterectomy, *n* (%)	860 (32)	0
Local or systemic oestrogen therapy, *n* (%)	2,206 (83)	4 (0.1)
Compartment of surgery, *n* (%)		49 (1.8)
Anterior^a^	537 (20)	
Apical^b^	1,599 (61)	
Posterior^c^	492 (19)	
Type of surgery, *n* (%)		0
Native tissue repair	2,166 (81)	
Transvaginal mesh	321 (12)	
Abdominal mesh^d^	190 (7.1)	

*SD* standard deviation, *BMI* body mass index, *POP-Q* Pelvic Organ Prolapse Quantification, *IQR* interquartile range, *Ba* most distal point of the anterior vaginal segment (0 indicates the hymenal level), *C* most distal point of the apical compartment, *Bp* most distal point of the posterior vaginal segment, *POP* pelvic organ prolapse

^a^Women with a vaginal procedure for anterior wall prolapse ± vaginal posterior repair

^b^Women with any procedure for apical compartment ± anterior and/or posterior repair

^c^Women with a vaginal procedure for posterior wall prolapse only

^d^Laparoscopic sacrocolpopexy, *n* = 173, open sacrocolpopexy, *n* = 17

The independent variables for the prognostic factor analyses were selected based on previous literature or clinical interest. These included age; body mass index (BMI); vaginal parity; smoking status; diabetes; history of hysterectomy and POP surgery; degree of anterior (continuous Ba), apical (C), and posterior (Bp) compartment prolapse; baseline SUI symptom (bother score 0–4); baseline urinary urge incontinence symptom (bother score 0–4); type of surgery (native tissue repair, transvaginal mesh, or abdominal mesh); and compartment of surgery. The categories for the compartment of surgery were:

1. Anterior group, i.e., procedures for the anterior compartment, with or without posterior compartment procedures, but no apical compartment procedures
2. Apical group, i.e., any procedure for the apical compartment with or without procedures for the anterior or posterior compartment
3. Posterior group, i.e., procedures for the posterior compartment only.

Multivariable models were fitted entering all relevant independent variables for each statistical question. Strong collinearity, as indicated by a Spearman’s correlation coefficient greater than 0.4, was observed between a history of POP surgery, a history of hysterectomy, and transvaginal mesh surgery. Therefore, these factors were not included in the same statistical model. Additionally, when the compartment of surgery was included in the model, POP-Q points were excluded.

## Results

### Study Flow and Characteristics of the Study Population

The selection of the study population is presented in Fig. 1. The study population (*N* = 2,677) had a mean age of 64 years, a mean BMI of 27 kg/m^2^, and a median vaginal parity of 2 (Table 1). Race was not recorded, but almost all the women were white. A quarter had a history of previous POP surgery. The majority of participants underwent native tissue repair (81%), while 12% had transvaginal mesh surgeries, and 7% underwent sacrocolpopexies. (Table 1) More comprehensive details about the surgical procedures in the FINPOP cohort have been previously published [7].

Symptom questionnaire data, including SUI symptom status, were available for 2,231 (84%) women at six months and 2,092 (79%) women at two years. The 22 women (0.8%) who underwent concomitant SUI surgery were excluded from the follow-up analyses (Fig. 1).

### Baseline SUI Symptoms

At baseline, 50% of participants reported SUI of any degree of bother, and 15% reported bothersome SUI (Table 2). Younger age, higher BMI, diabetes, and urge urinary incontinence symptoms were associated with increased symptom bother at baseline. The severity of SUI symptoms did not correlate with the degree of anterior or posterior prolapse, but a weak inverse relationship was found between the severity of SUI and the degree of apical prolapse (Tables 2 and 3).

**Table 2:** Prevalence of stress urinary incontinence at baseline

		*N*	Any degree of bother	Bothersome symptom
Total population		2,677	1,329 (50)	391 (15)
Overall stage	0–1	15	7 (47)	3 (20)
	2	1,231	644 (52)	199 (16)
	3–4	1,306	623 (48)	174 (13)
Anterior stage	0–1	686	330 (48)	100 (15)
	2	1,109	579 (52)	174 (16)
	3–4	796	388 (49)	109 (14)
Apical stage	0–1	1,244	650 (52)	187 (15)
	2	866	427 (49)	133 (15)
	3–4	446	204 (46)	55 (12)
Posterior stage	0–1	1,084	535 (49)	156 (14)
	2	1,111	565 (51)	173 (16)
	3–4	386	187 (48)	51 (13)
Age	<55	560	290 (52)	133 (24)
	55–75	1,750	851 (49)	216 (12)
	>75	365	188 (52)	42 (12)
BMI	<25	940	422 (45)	115 (12)
	25–30	1,132	563 (50)	162 (14)
	>30	519	302 (58)	105 (20)
DM	Yes	249	150 (60)	45 (18)
	No	2,428	1,179 (49)	346 (14)

*BMI* body mass index, *DM* diabetes mellitus

Values given as number (percentage). Stage of prolapse is given according to the simplified Pelvic Organ Prolapse Quantification (POP-Q), stages 0–1 and 3–4 combined, as the vaginal length was not measured

**Table 3:** Factors associated with baseline stress urinary incontinence

Variable	OR^a^	Adjusted OR^b^
Age (years)	**0.99 (0.99–0.999)**	**0.98 (0.97–0.99)**
BMI (kg/m^2^)	**1.05 (1.03–1.07)**	**1.03 (1.01–1.05)**
Ba (cm, anterior wall prolapse)	1.00 (0.96–1.03)	0.99 (0.95–1.04)
C (cm, apical prolapse)	**0.97 (0.94–0.99)**	**0.95 (0.92–0.97)**
Bp (cm, posterior wall prolapse)	1.00 (0.96–1.03)	1.04 (1.00–1.09)
Vaginal parity	**1.06 (1.01–1.12)**	1.03 (0.97–1.10)
Smoking (Ref. no smoking)	1.06 (0.82–1.37)	1.22 (0.91–1.65)
DM (Ref. no DM)	**1.52 (1.20–1.93)**	**1.40 (1.05–1.85)**
Prior POP surgery (Ref. no prior)	0.97 (0.82–1.15)	0.99 (0.81–1.20)
Prior hysterectomy (Ref. no prior)	0.97 (0.83–1.13)	0.93 (0.78–1.12)
Baseline UUI bother score	**2.13 (2.00–2.27)**	**2.21 (2.06–2.37)**

*OR* odds ratio, *BMI* body mass index, *DM* diabetes mellitus, *Ref.* reference category, *POP* pelvic organ prolapse, *UUI* urgency urinary incontinence

Generalized linear models (ordinal logistic), baseline ordinal bother score (scale 0–4; 0 – no symptom, 4 – symptom quite a bit bothersome) as dependent variable (ascending)

Statistically significant associations are bolded

^a^Univariate analysis

^b^Multivariable analysis. When history of prior hysterectomy was included in the model, prior POP surgery was not included (strong collinearity)

### Changes in SUI Symptoms after POP Surgery (Without a Continence Procedure)

#### Women with Baseline SUI

Among women with pre-existing SUI, 35% reported complete symptom resolution, and an additional 14% reported improvement 2 years after surgery without any SUI procedures. Sixty-five percent continued to experience persistent symptoms; 15% reported worsening of their symptoms (Table 4). The average severity of SUI decreased after surgery for all vaginal compartments (*p* < 0.001 for each surgical compartment).

**Table 4:** Changes in stress urinary incontinence (*SUI*) symptoms after pelvic organ prolapse surgery

	Baseline	Change at follow-up	6 months^a^	2 years^b^	SUI procedures^c^
Total population, *N* = 2,677	Incontinent, *n* = 1,329 (50)	Improved	607/1,092 (56)	494/1,005 (49)	67/1,308 (5.1)
		Resolved	428/1,092 (39)	354/1,005 (35)	
		Same	407/1,092 (37)	360//1,005 (36)	
		Worse	78/1,092 (7.1)	151/1,005 (15)	
	Continent, *n* = 1,348 (50)	De Novo, any bother	166/1,139 (15)	218/1,087 (20)	11/1,347 (0.8)
		De Novo, bothersome	18/1,139 (1.6)	35/1,087 (3.2)	
Anterior, *N* = 537	Incontinent, *n* = 285 (53)	Resolved	78/229 (34)	57/205 (28)	15/285 (5.3)
	Continent, *n* = 252 (47)	De Novo, any bother	33/215 (15)	42/209 (20)	1/252 (0.4)
		De Novo, bothersome	2/215 (0.9)	5/209 (2.4)	
Apical, *N* = 1,599	Incontinent, *n* = 766 (48)	Resolved	275/651 (42)	239/608 (39)	36/766 (4.7)
	Continent, *n* = 833 (52)	De Novo, any bother	107/706 (15)	134/670 (20)	8/833 (1.0)
		De Novo, bothersome	14/706 (2.0)	22/670 (3.3)	
Posterior, *N* = 492	Incontinent, *n* = 242 (49)	Resolved	73/200 (37)	54/179 (30)	14/242 (5.8)
	Continent, *n* = 250 (51)	De Novo, any bother	25/211 (12)	40/198 (20)	2/250 (0.8)
		De Novo, bothersome	2/211 (0.9)	8/198 (4.0)	
NTR, *N* = 2,166	Incontinent, *n* = 1,074 (50)	Resolved	346/877 (40)	267/794 (34)	47/1,055 (4.5)
	Continent, *n* = 1,092 (50)	De Novo, any bother	107/918 (12)	160/870 (18)	6/1,091 (0.5)
		De Novo, bothersome	8/918 (0.9)	21/870 (2.4)	
TVM, *N* = 321	Incontinent, *n* = 166 (52)	Resolved	46/138 (33)	54/138 (39)	17/164 (10)
	Continent, *n* = 155 (48)	De Novo, any bother	35/132 (27)	45/133 (34)	3/155 (1.9)
		De Novo, bothersome	7/132 (5.3)	10/133 (7.5)	
AM, *N* = 190	Incontinent, *n* = 89 (47)	Resolved	36/77 (47)	33/73 (45)	3/89 (3.4)
	Continent, *n* = 101 (53)	De Novo, any bother	24/89 (27)	13/84 (16)	2/101 (2.0)
		De Novo, bothersome	3/89 (3.4)	4/84 (4.8)	
Age < 55, *N* = 560	Incontinent, *n* = 290 (52)	Resolved	88/219 (40)	57/194 (29)	18/279 (6.5)
	Continent, *n* = 270 (48)	De Novo, any bother	23/220 (11)	25/197 (13)	0 (0)
		De Novo, bothersome	3/220 (1.4)	6/197 (3.0)	
Age 55–75, *N* = 1,750	Incontinent, *n* = 851 (49)	Resolved	301/719 (42)	262/677 (39)	38/842 (4.5)
	Continent, *n* = 899 (51)	De Novo, any bother	109/768 (14)	147/746 (20)	8/898 (0.9)
		De Novo, bothersome	12/768 (1.6)	22/746 (2.9)	
Age > 75, *N* = 365	Incontinent, *n* = 188 (52)	Resolved	39/154 (25)	35/134 (26)	11/187 (5.9)
	Continent, *n* = 177 (49)	De Novo, any bother	34/149 (23)	46/143 (32)	3/177 (1.7)
		De Novo, bothersome	3/149 (2.0)	7/143 (4.9)	

*NTR* native tissue repair, *TVM* transvaginal mesh, *AM* abdominal mesh

Presented for the total population and stratified for the compartment of surgery, type of surgery, and age. Values are given as number/Number (%). For women who received a surgical procedure for SUI during the follow-up, a bother score of 4 (maximum bother) was re-coded after the date of the SUI procedure

^a^Symptom change from baseline to 6 months, calculated for cases with data available for baseline and 6 months

^b^Symptom change from baseline to 24 months, calculated for cases with data available for baseline and 24 months

^c^Number of SUI procedures during the 2-year follow-up, calculated for the entire study population

Sixty-seven women (5%) underwent at least one procedure for persistent SUI during the 2-year follow-up period (59 mid-urethral slings, 9 periurethral bulking; Table 4). The median time from the index operation was 264 days (interquartile range [IQR] 185–466).

**Fig. 2:**
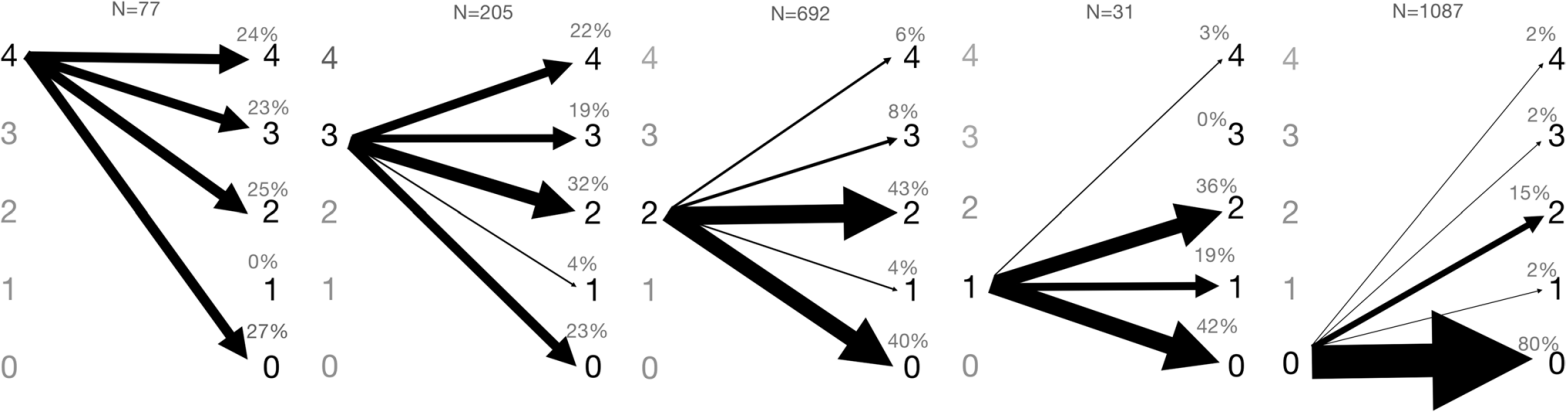
Change in stress urinary incontinence symptom severity from baseline to 2 years. Each image depicts change in symptom severity from baseline to 2 years’ follow-up stratified by baseline symptom severity: baseline symptom severity on the left and 2-year symptom severity on the right. The scale of symptom severity: 4—symptom quite a bit bothersome; 3—symptom moderately bothersome; 2—symptom somewhat bothersome; 1—symptom present but not at all bothersome; 0—symptom not present. The thickness of the arrow is proportional to the percentage

Women with strong baseline SUI symptoms were more likely to experience persistent SUI and to need subsequent SUI procedures than those with milder symptoms (Table 5, univariate analysis; Table 6, multivariable analysis). Although 40% of women with mild baseline SUI (score 1–2; 286 out of 723) achieved complete symptom resolution 2 years after surgery, only 24% of those with bothersome baseline SUI (score 3–4; 68 out of 282) reported complete resolution and 43% (120 out of 282) still reported bothersome SUI (Fig. 2). SUI procedures were performed on 3% of women (30 out of 933) with mild baseline SUI and on 10% (37 out of 375) of those with bothersome baseline SUI during the 2-year follow-up.

**Table 5:** Factors associated with persistent stress urinary incontinence (*SUI*), procedures for persistent SUI, and de novo SUI symptoms. Univariate analysis

Prognostic factor	Persistent SUI 2 years, OR^a^	Procedure for persistent SUI, OR^b^	De novo SUI 6 months, OR^a^	De novo SUI 2 years OR^a^
Baseline SUI bother score	**2.13 (1.78–2.56)**	**2.04 (1.50–2.79)**	N/A	N/A
Age (years)	0.99 (0.98–1.00)	0.99 (0.97–1.01)	**1.03 (1.02–1.05)**	**1.03 (1.02–1.05)**
BMI (kg/m^2^)	**1.04 (1.01–1.07)**	1.01 (0.95–1.08)	1.04 (1.00–1.08)	1.04 (1.00–1.07)
Ba (anterior wall)	0.99 (0.94–1.05)	1.01 (0.89–1.14)	1.07 (0.98–1.15)	**1.08 (1.01–1.16)**
C (apex)	**0.92 (0.88–0.95)**	**0.88 (0.81–0.96)**	1.00 (0.95–1.06)	1.03 (0.98–1.08)
Bp (posterior wall)	1.00 (0.95–1.07)	0.93 (0.81–1.06)	1.01 (0.93–1.09)	1.01 (0.94–1.09)
Vaginal parity	1.04 (0.95–1.13)	0.91 (0.74–1.14)	0.89 (0.78–1.03)	1.00 (0.90–1.11)
DM	1.12 (0.78–1.61)	1.23 (0.60–2.54)	1.41 (0.79–2.53)	**1.65 (1.00–2.73)**
Smoking	0.87 (0.57–1.32)	0.66 (0.24–1.86)	0.91 (0.48–1.70)	0.75 (0.42–1.32)
Prior POP surgery	0.87 (0.67–1.14)	1.14 (0.65–1.98)	**1.98 (1.40–2.81)**	**1.45 (1.05–2.01)**
Prior hysterectomy	0.91 (0.72–1.16)	1.39 (0.83–2.34)	**1.81 (1.30–2.53)**	**1.40 (1.03–1.91)**
Baseline UUI bother score	**1.25 (1.14–1.37)**	**1.34 (1.08–1.66)**	**1.24 (1.09–1.41)**	**1.31 (1.17–1.47)**
Type of surgery				
NTR	1 (Ref.)	1 (Ref.)	1 (Ref.)	1 (Ref.)
TVM	0.84 (0.60–1.18)	**2.48 (1.39–4.44)**	**2.79 (1.81–4.30)**	**2.3 (1.56–3.42)**
AM	0.75 (0.47–1.18)	0.75 (0.23–2.45)	**2.77 (1.67–4.59)**	0.86 (0.46–1.58)
Compartment of surgery				
Anterior	1 (Ref.)	1 (Ref.)	1 (Ref.)	1 (Ref.)
Apical	**0.61 (0.46–0.81)**	0.89 (0.45–1.65)	0.99 (0.65–1.51)	1.00 (0.68–1.47)
Posterior	0.91 (0.63–1.30)	1.11 (0.52–2.34)	0.74 (0.42–1.29)	1.04 (0.64–1.69)

*OR* odds ratio, *NA* not applicable, *BMI* body mass index, *DM* diabetes mellitus, *POP* pelvic organ prolapse, *UUI* urgency urinary incontinence, *NTR* native tissue repair, *TVM* transvaginal mesh, *AM* abdominal mesh, *Ref.* reference category

Results from univariate analyses, odds ratios are shown. Statistically significant associations are bolded

^a^Generalized linear models (ordinal logistic); ordinal bother score (scale 0–4; 0 – no symptom, 4 – symptom quite a bit bothersome) as dependent variable (ascending)

^b^Binary logistic regression; subsequent SUI procedure for persistent SUI during 2-year follow-up as dependent variable

**Table 6:** Factors associated with persistent stress urinary incontinence (*SUI*) symptoms, procedures for persistent SUI, and de novo SUI symptoms. Multivariable analysis

Prognostic factor	Persistent SUI symptom 2 years, aOR^a^	Procedure for persistent SUI, aOR^b^	De novo SUI 6 months, aOR^a^	De novo SUI 2 years, aOR^a^
Baseline SUI bother score	**2.04 (1.65–2.53)**	**1.91 (1.27–2.86)**	N/A	N/A
Age (years)	1.00 (0.98–1.01)	0.98 (0.96–1.01)	**1.03 (1.01–1.05)**	**1.03 (1.01–1.05)**
BMI (kg/m^2^)	1.02 (0.99–1.06)	1.01 (0.94–1.07)	**1.05 (1.001–1.09)**	1.02 (0.98–1.06)
Ba (anterior wall)	1.05 (0.98–1.13)	1.08 (0.91–1.27)	1.05 (0.95–1.16)	1.02 (0.93–1.12)
C (apex)	**0.89 (0.85–0.93)**	**0.85 (0.76–0.94)**	0.96 (0.90–1.02)	1.01 (0.96–1.07)
Bp (posterior wall)	1.01 (0.95–1.08)	0.97 (0.82–1.13)	1.07 (0.97–1.18)	1.05 (0.97–1.15)
Vaginal parity	0.96 (0.88–1.06)	0.88 (0.69–1.11)	0.93 (0.79–1.08)	1.04 (0.93–1.16)
DM	1.13 (0.75–1.70)	1.21 (0.52–2.81)	1.58 (0.83–3.01)	1.61 (0.91–2.83)
Smoking	0.77 (0.49–1.22)	0.47 (0.14–1.58)	1.13 (0.54–2.39)	0.73 (0.36–1.47)
Prior POP surgery	1.02 (0.67–1.56)	0.69 (0.23–2.07)	1.01 (0.53–1.93)	0.84 (0.47–1.52)
Prior hysterectomy	0.75 (0.52–1.07)	0.47 (0.18–1.23)	0.93 (0.53–1.64)	1.08 (0.65–1.80)
Baseline UUI bother score	1.07 (0.95–1.19)	1.08 (0.84–1.40)	**1.19 (1.03–1.37)**	**1.21 (1.06–1.38)**
Type of surgery				
NTR	1 (Ref.)	1 (Ref.)	1 (Ref.)	1 (Ref.)
TVM	0.90 (0.62–1.30)	**2.80 (1.40–5.59)**	**2.12 (1.29–3.48)**	**1.93 (1.24–3.00)**
AM	1.12 (0.68–1.84)	1.52 (0.43–5.36)	**2.83 (1.57–5.13)**	0.72 (0.35–1.47)
Compartment of surgery				
Anterior	1 (Ref.)	1 (Ref.)	1 (Ref.)	1 (Ref.)
Apical	**0.58 (0.43–0.80)**	0.73 (0.35–1.49)	0.77 (0.47–1.27)	0.98 (0.63–1.53)
Posterior	0.93 (0.64–1.37)	1.32 (0.58–2.96)	1.01 (0.55–1.83)	1.36 (0.80–2.30)

*aOR* adjusted odds ratio, *N/A* not applicable, *BMI* body mass index, *DM* diabetes mellitus, *POP* pelvic organ prolapse, *UUI* urgency urinary incontinence, *NTR* native tissue repair, *TVM* transvaginal mesh, *AM* abdominal mesh, *Ref.* reference category

Results from multivariable analyses, adjusted odds ratios are shown. ﻿Statistically significant associations are bolded

^a^Generalized linear models (ordinal logistic); ordinal bother score (scale 0–4; 0 – no symptom, 4 – symptom quite a bit bothersome) as dependent variable (ascending)

^b^Binary logistic regression; subsequent SUI procedure for persistent SUI during 2-year follow-up as dependent variable

Explanatory variables included in the primary multivariable models: (baseline SUI bother score), age, BMI, Ba, C, Bp, vaginal parity, DM, smoking, baseline UUI bother score, and type of surgery. When the compartment of surgery was included in the model, Ba, C, and Bp were removed. When prior POP surgery and prior hysterectomy were entered into the model (one at a time), type of surgery was removed, and patients undergoing transvaginal or abdominal mesh surgery were excluded from the analysis owing to strong collinearity

In contrast, a good outcome was more likely among women with advanced apical prolapse at baseline and those undergoing apical compartment surgery (Tables 4, 5, and 6).

Women who underwent transvaginal mesh surgery were at an increased risk of undergoing subsequent SUI procedures for persistent SUI, even though the risk of persistent SUI symptoms itself was not increased (Tables 4, 5, and 6). None of the remaining patient characteristics were found to be associated with persistent SUI (Tables 5 and 6).

#### Women without Baseline SUI

Among women without baseline SUI symptoms, 20% had developed de novo SUI symptoms of any degree and 3% had developed SUI symptoms of bothersome degree by the 2-year follow-up (Table 4). Eleven women (1%) underwent a procedure for de novo SUI (10 mid-urethral slings, 1 periurethral bulking; Table 4). The median time from the index operation was 272 days (IQR 173–425).

Neither the compartment nor the degree of prolapse was associated with de novo SUI symptoms in the multivariable model. Older women, women who underwent transvaginal mesh surgery, and women with baseline urgency urinary incontinence symptoms were at an increased risk of developing de novo SUI at 6 and 24 months. Obesity and abdominal mesh surgery were found to be associated with de novo SUI at 6 months, but not at 24 months (Tables 4, 5, and 6).

## Discussion

### Main Findings

This nationwide observational study explored changes in SUI symptoms over a 2-year period following POP surgery without concomitant SUI intervention, aiming to understand the impact of postoperative SUI on daily life and identify predictive factors.

Half of the women undergoing POP surgery reported pre-existing SUI. Symptoms resolved or improved after POP surgery alone in half of the cases, and only 5% underwent a procedure for persistent SUI. Improvement was observed after surgery on any compartment, with slightly better outcomes noted in surgeries involving the apical compartment. Severe baseline symptoms predicted persistent SUI, whereas no other variables studied were associated with symptom persistence.

A fifth of the preoperatively continent women developed de novo SUI symptoms, but only 3% experienced bothersome symptoms, and 1% underwent a procedure for it. De novo symptoms occurred irrespective of the compartment or degree of POP. Older age, transvaginal mesh surgery, and baseline urgency urinary incontinence predisposed women to de novo SUI symptoms at both 6 and 24 months after surgery.

### Results in the Context of What is Known

International guidelines advocate for the combination of POP and SUI surgery in women with overt or occult preoperative SUI, supported by RCT evidence [12]. However, global rates of concomitant SUI surgery vary widely, ranging from 0.2% in Denmark and 0.8% in this study to 36% in the USA [13, 14]. This variability highlights the limited universal acceptance of this recommendation and reflects diverse patient or surgeon preferences. Limited access to mesh or concerns related to mesh may increase interest in the staged approach, as indicated by the declining rate of concomitant SUI surgery in the USA [14].

In daily practice, a staged strategy results in significantly fewer subsequent SUI procedures than RCTs suggest [6]. We found a 5% risk of subsequent SUI procedures in preoperatively incontinent women and a 1% risk in preoperatively continent women, contrasting with 40% and 6% risks respectively reported in a systematic review [6]. Similar disparity was observed in a recent Danish historical cohort study (*N* = 15,832), which reported risks of 12% and 2% respectively during the 10 years following POP surgery [13]. The number needed to treat (NNT) with a concomitant mid-urethral sling to prevent one subsequent sling in pre-existing SUI is 2.5 according to RCTs. In our population-based cohort, the NNT settles at around 20. This difference likely arises from RCTs including a SUI procedure in the protocol, whereas in real life, further treatment occurs only if women specifically seek it.

A third of women with pre-existing SUI achieved complete symptom resolution after POP surgery alone, consistent with previous literature (29–52%) [1, 15–18]. For these women, additional SUI surgery would have been unnecessary. On the other hand, two-thirds experienced persistent SUI, but only 5% underwent subsequent SUI procedures. Financial barriers are unlikely to explain this, as public health care covers expenses in Finland. It is more likely that some women do not expect complete dryness, as supported by a Norwegian RCT, where 21% of women with persistent SUI symptoms declined the planned staged SUI surgery owing to a lack of bother [16]. Cultural factors, surgeons’ preferences, follow-up practices, and concerns about complications may also influence decision-making. Notably, in our study, transvaginal mesh surgery was associated with a higher number of subsequent SUI procedures, despite comparable rates of persistent SUI symptoms, possibly due to patient or surgeon preferences or more rigorous follow-up.

Our observation that high baseline symptom severity is a risk factor for persistent SUI symptoms finds support in a Danish database study (*n* = 1,657) with a short, 3-month follow-up [15]. Another study (*n* = 93) did not find this association, but wide CIs indicate a lack of statistical power [18]. Both our study and the Danish study suggest that improved SUI symptoms could be observed after surgery on any vaginal compartment [15]. Additionally, in a Swedish RCT, perineorrhaphy resolved pre-existing SUI symptoms in 44% of women, whereas physiotherapy showed no significant improvement [19]. The biomechanical rationale behind SUI improvement after posterior prolapse repair is not evident. It is possible that pelvic floor muscle function improves when the bulging mass is removed or the reconstructed perineal body provides support for the urethra.

The incidence of de novo SUI varies greatly in the existing literature (4–49%), owing to differences in baseline incontinence status, surgical techniques, the definitions, and follow-up duration [1–5, 20]. In line with our finding of a 20% rate at 2 years, a prospective cohort study from the Netherlands reported a 22% rate of de novo SUI symptoms of any degree at 1 year [1]. However, clinically meaningful de novo SUI is likely better reflected by the rate of bothersome symptoms (3% in this study) or the need for surgical intervention for de novo SUI (1%).

Our finding of consistent de novo SUI symptom rates irrespective of the surgical compartment and degree of prolapse is unexpected. A prevalent theory suggests that advanced anterior prolapse may cause urethral kinking, potentially masking SUI. When this kinking is relieved during POP surgery, de novo SUI may emerge [21]. Consequently, one could anticipate large and anterior dominant prolapses to increase the de novo SUI risk. However, although one study with 164 patients undergoing sacrocolpopexy showed a correlation between the preoperative degree of anterior wall prolapse and de novo SUI [22], other studies support our findings [4, 23, 24]. The interplay between anatomy and incontinence appears to be complex, with bladder neck anatomy possibly more pivotal than cystocele size [25]. Additionally, posterior POP may exert external pressure on the urethra rather than cause urethral kinking. If the continence mechanism is compromised, removing this compression during prolapse surgery could unmask SUI. This notion is supported by a UK study, which found that the lower the descent of the posterior wall after sacrocolpopexy, the lower the incidence of de novo SUI [26].

Accurately predicting de novo SUI has proven to be a challenge. Although occult SUI (i.e., ﻿sign of SUI observed only during prolapse reduction) is used as a predictor, its diagnostic accuracy varies across studies [27], and one of the largest studies indicated only a slight improvement over chance [23]. Consequently, it is not a standard practice in Finland. A risk calculator that incorporates stress test results and various patient characteristics [23] offers moderate performance at best, with an area under the curve or concordance index ranging from 0.50 to 0.69 in external validation studies [27–29]. Its limited accuracy is understandable, given the weak, non-existent, and even conflicting correlations between its factors and de novo SUI. For example, both our study and a study by Lo et al. identified an increased risk of de novo SUI with advancing age [24], whereas the study on which the calculator was based reported the opposite result [23].

### Clinical Implications

Based on our data, women planning to undergo POP surgery with bothersome SUI symptoms can be informed that without concomitant SUI surgery, they have a 24% chance of complete symptom resolution and a 43% chance of persistent bothersome symptoms, on average. If they are unwilling to accept the higher risk of adverse events associated with a concomitant continence procedure and are comfortable with the possibility of undergoing secondary surgery later, a staged strategy may be appropriate.

As bothersome de novo SUI symptoms develop infrequently and their prediction is difficult, a staged SUI treatment strategy seems a viable option for continent women. Comprehensive preoperative counseling is crucial to prevent misunderstandings should distressing de novo SUI develop.

### Strengths and Limitations

The strengths of this study include the large, nationwide population, pragmatic setting, prospective data collection, use of a validated questionnaire, ﻿and the duration of follow-up. Furthermore, the standard treatment practice in Finland, where the staged strategy is preferred regardless of the preoperative continence status, allowed for the assessment of the impact of prolapse surgery itself.

This study has limitations. The absence of a comparison group hampers the ability to attribute improvements solely to the specific effects of surgery; nonspecific factors such as regression to the mean and the natural progression of the disease may have influenced the outcomes. Additionally, information regarding conservative SUI treatments and repeat POP operations during the follow-up was unavailable. Objective data, such as cough stress tests, frequency volume charts, and urodynamic studies, were lacking, potentially leading to misclassification between SUI and urgency urinary incontinence. Furthermore, there is a possibility that SUI developing against a background of urgency urinary incontinence involves cough-provoked detrusor overactivity. However, a previous report found that responses indicating bothersome SUI in the PFDI-20 (item 17) demonstrate a strong correlation with urodynamic SUI [30]. Although the loss of follow-up was at an acceptable level, it is important to note that this may not occur completely at random. Treatment satisfaction could impact the willingness to reply, potentially introducing bias into the results concerning symptom changes. Nevertheless, the number of subsequent SUI procedures remains unbiased, as these data were available for all except one participant. The clinical evaluation by multiple doctors may have introduced heterogeneity into the POP-Q measurements. Finally, although the population-based setting ensures diverse patients, surgical techniques, and surgeons, thereby enhancing external validity, the predominantly white and culturally homogenous study population may limit the generalizability of the results to other ethnic groups.

## Conclusion

As patient goals and preferences may vary, the SUI treatment strategy during POP surgery should be based on informed decisions. Our results are valuable for preoperative counseling. According to these findings, POP surgery alone is sufficient to address SUI symptoms for many patients. Accurately predicting individuals who will require staged SUI procedures appears impossible, but our data imply that the risk is lower than prior trials have indicated. Future research should explore the reasons why some women do not undergo staged procedures for bothersome postoperative SUI.
